# Serum Uric Acid Concentrations in Meat Eaters, Fish Eaters, Vegetarians and Vegans: A Cross-Sectional Analysis in the EPIC-Oxford Cohort

**DOI:** 10.1371/journal.pone.0056339

**Published:** 2013-02-13

**Authors:** Julie A. Schmidt, Francesca L. Crowe, Paul N. Appleby, Timothy J. Key, Ruth C. Travis

**Affiliations:** Cancer Epidemiology Unit, Nuffield Department of Medicine, University of Oxford, Oxford, United Kingdom; The University of Texas M. D. Anderson Cancer Center, United States of America

## Abstract

**Introduction:**

Circulating concentrations of uric acid may be affected by dietary components such as meat, fish and dairy products, but only a few studies have compared uric acid concentrations among individuals who exclude some or all of these foods from their diet. The aim of this study was to investigate differences in serum uric acid concentrations between meat eaters, fish eaters, vegetarians and vegans.

**Subjects and Methods:**

A sample of 670 men and 1,023 women (424 meat eaters, 425 fish eaters, 422 vegetarians and 422 vegans, matched on age and sex) from the European Prospective Investigation into Cancer and Nutrition Oxford cohort were included in this cross-sectional analysis. Diet was assessed using a semi-quantitative food frequency questionnaire and serum concentrations of uric acid were measured. Mean concentrations of uric acid by diet group were calculated after adjusting for age, body mass index, calcium and alcohol intake.

**Results:**

In both men and women, serum uric acid concentrations differed significantly by diet group (p<0.0001 and p = 0.01, respectively). The differences between diet groups were most pronounced in men; vegans had the highest concentration (340, 95% confidence interval 329–351 µmol/l), followed by meat eaters (315, 306–324 µmol/l), fish eaters (309, 300–318 µmol/l) and vegetarians (303, 294–312 µmol/l). In women, serum uric acid concentrations were slightly higher in vegans (241, 234–247 µmol/l) than in meat eaters (237, 231–242 µmol/l) and lower in vegetarians (230, 224–236 µmol/l) and fish eaters (227, 221–233 µmol/l).

**Conclusion:**

Individuals consuming a vegan diet had the highest serum concentrations of uric acid compared to meat eaters, fish eaters and vegetarians, especially in men. Vegetarians and individuals who eat fish but not meat had the lowest concentrations of serum uric acid.

## Introduction

Uric acid is the end product of purine metabolism, generated from the breakdown of DNA, RNA and ATP [1]. The ability to further metabolise uric acid has been lost in humans due to two mutations that silence the gene coding for the enzyme uricase, which can further degrade uric acid. Therefore, humans are prone to a high concentration of serum uric acid. High circulating concentrations of uric acid can lead to gout, a common form of arthritis [1], and have also been linked to chronic kidney disease [2], cardiovascular disease [3], [4] and cancer [5]–[7]. However, given that uric acid is also related to a number of other factors such as age and body mass index (BMI), the causal nature of these associations is not clear [6], [8].

High uric acid concentrations can result from low rates of excretion, primarily through the kidneys, and from overproduction of uric acid due to an excess of purine precursors from synthesis, cell turnover and diet [1]. Certain dietary components are thought to affect concentrations of uric acid. For instance, meat and fish may increase the concentration of uric acid because of the high purine content of these foods [9], [10], and dairy products may lower uric acid concentrations [10], [11] by increasing the excretion of uric acid and its precursor xanthine [11]. Thus, individuals who avoid consuming one or more of these foods groups might be expected to have different circulating concentrations of uric acid. Some small studies have observed a lower concentration of uric acid in vegetarians compared to meat eaters [12]–[14]. However, none of these studies clearly differentiated between meat eaters and fish eaters or between vegetarians and vegans. Therefore, the aim of this study was to investigate differences in the concentration of uric acid between meat eaters, fish eaters, vegetarians and vegans in the Oxford arm of the European Prospective Investigation into Cancer and Nutrition (EPIC-Oxford).

## Materials and Methods

### Study population

The EPIC-Oxford cohort includes 65,429 men and women aged 20 years or older who were recruited from around the United Kingdom between 1993 and 1999. The study was designed to investigate diet, lifestyle and risk of cancer among people with different dietary habits and thus aimed to recruit vegetarians and vegans as well as participants from the general population. A detailed description of the recruitment process has been published elsewhere [15]. In brief, participants from the general population were recruited through general practice surgeries, whilst postal recruitment was targeted to recruit a large number of vegetarians and vegans but also resulted in a high number of non-vegetarians. In the current study, 71%, 97%, 99% and 100% of meat eaters, fish eaters, vegetarians and vegans, respectively, were recruited via post. The protocol for the EPIC-Oxford study was approved by a multi-centre research ethics committee (MREC/02/0/90), now called “Scotland A Research Ethics Committee”, and all participants gave written informed consent.

The present cross-sectional analysis includes men and women who (i) had provided a blood sample at recruitment, (ii) had a known smoking and diet group, (iii) had responded to ≥80% of the relevant questions in the FFQ (130 questions for meat eaters and fish eaters, and 113 questions for vegetarians and vegans) and had an energy intake between 3.3 and 16.7 MJ (800–4,000 kcal) for men or between 2.1 and 14.7 MJ (500–3,500 kcal) for women, (iv) did not have prior cancer (excluding non-melanoma skin cancer) or cardiovascular disease, (v) were not receiving treatment for any long-term illness or condition, (vi) were not pregnant or taking oral contraceptives or hormone therapy for menopause (women only), and (vii) were younger than 90 years at time of blood collection. In order to maximise the heterogeneity of dietary exposure, approximately equal numbers of participants in each of the four diet groups were randomly selected from participants who were stratified by sex and by 10 year age categories. This resulted in 424 meat eaters, 425 fish eaters, 422 vegetarians and 422 vegans being included in this study.

### Assessment of diet and lifestyle

At recruitment, participants completed a validated semi-quantitative food frequency questionnaire [16], [17] (FFQ) with additional questions relating to prior disease, anthropometry and lifestyle factors such as smoking.

Participants were categorised into one of four diet groups based on their answers to the questions: “Do you eat any (i) meat, (ii) fish, (iii) dairy products and (iv) eggs?” The derived diet groups were: meat eaters, fish eaters (do not eat meat but do eat fish), vegetarians (do not eat meat or fish) and vegans (do not eat meat, fish, dairy products or eggs).

In the FFQ, participants were asked to report their average intake of 130 food items over the preceding 12 months in nine categories ranging from “Never or less than once per month” to “6 or more times per day”. The mean daily intakes of food items were estimated by multiplying the frequency of consumption by a specified portion size (mostly taken from Ministry of Agriculture, Fisheries and Food, Food portion sizes [18]). The mean nutrient intake was estimated by multiplying the amount of food consumed by the nutrient content of the food item (mainly based on the fifth edition of McCance and Widdowson's The Composition of Foods and its supplements [19]–[28]). The individual food items were categorised into food groups where appropriate, e.g. total meat (red meat, processed meat, liver and poultry) and fructose-rich drinks (fruit juice and sugar sweetened soft drinks).

In the questionnaire, all participants were asked to report their weight and height. Weight and height were also measured in a sub-sample (n = 4,808) of the cohort, and self-reported data showed good agreement with the measured data (r>0.9) [29]. Either self-reported or measured height and weight were used to calculate body mass index (BMI; weight (kg)/(height (m))^2^). Smoking was categorised as “never”, “former”, “current light” (<15 cigarettes/day) and “current heavy” (≥15 cigarettes/day), and age of the participants was recorded at time of blood collection.

### Laboratory method

At recruitment or shortly after, participants attended their local general practice surgeries where a blood sample was taken (participants were not required to fast). Blood was transported overnight to a laboratory in Norfolk by mail at ambient temperature, where samples were centrifuged and serum was aliquoted into 0.5 ml plastic straws. These were heat-sealed at both ends and stored in liquid nitrogen (−196°C) until 2011 and subsequently in electric freezers (−80°C) until analysis. A Beckman Synchron DxC autoanalyser (Beckman Coulter, High Wycombe, UK) was used to measure serum uric acid in 2011. Pooled serum samples (n = 196) were included in each run (blinded) and the overall coefficient of variation for uric acid was 0.9%.

### Statistical analysis

All analyses were performed for men and women separately. The distributions of dietary and non-dietary characteristics were compared between the four diet groups; Pearson's χ^2^ test was used for categorical variables, and for continuous variables one-way ANOVA was used for normally distributed variables and the Kruskal-Wallis test for non-normally distributed variables.

In order to facilitate comparability between results with different levels of adjustment, participants with missing data on BMI were excluded (30 men and 34 women) leaving 408 meat eaters, 405 fish eaters, 404 vegetarians and 412 vegans for further analysis. Partial correlation coefficients between uric acid and dietary and non-dietary characteristics, respectively, were examined adjusting for age (20–29; 30–39; 40–49; 50–59; ≥60 years) and BMI (<20; 20–<22.5; 22.5–<25; 25–<27.5; ≥27.5 kg/m^2^).

Means and 95% confidence intervals (CI) of uric acid concentration were calculated for each diet group adjusted for age (20–29; 30–39; 40–49; 50–59; ≥60 years) and alcohol intake (sex-specific fifths) and then further for BMI (<20; 20–<22.5; 22.5–<25; 25–<27.5; ≥27.5 kg/m^2^) and calcium intake (sex-specific fifths) using multiple linear regression.

All p-values were two-sided and p<0.05 was considered statistically significant. All analyses were performed using the STATA statistical package version 12 (StataCorp., Texas, USA).

## Results

Characteristics and intake of food and nutrients for men and women subdivided by diet group are shown in Tables 1 and 2. The average age at blood collection was 45 years (standard deviation (SD) 11) in men and 40 years (SD 11) in women.

**Table 1:** Characteristics and intake of certain foods and nutrients by diet group among men.

	Meat eaters (n = 168)		Fish eaters (n = 168)		Vegetarians (n = 167)		Vegans (n = 167)		
Characteristics	Mean	SD	Mean	SD	Mean	SD	Mean	SD	p^1^
Age (years)	44.8	10.5	44.8	10.8	44.8	10.3	45.0	11.1	>0.9
BMI (kg/m^2^)^2^	24.9	3.1	23.2	2.9	23.3	2.6	22.4	3.2	<0.0001
Current smoking (n)^3^	23		16		8		10		0.1

	Median	IQR	Median	IQR	Median	IQR	Median	IQR	
Total meat (g/d)^4^	77.6	40.0–109.0	-	-	-	-	-	-	-
Fish (g/d)	29.4	19.8–41.8	29.5	15.7–47.3	-	-	-	-	0.7
Eggs (g/d)	7.0	3.5–21.5	7.0	3.5–21.5	7.0	0.5–21.5	-	-	0.1
Dairy milk (g/d)^2^	293	293–439	293	146–439	293	146–439	-	-	0.01
Dairy yoghurt (g/d)	18.9	2.5–55.4	18.9	10.1–63.0	35.3	10.1–63.0	-	-	0.05
Dairy cheese (g/d)	15.2	5.4–23.0	23.0	13.9–34.6	27.5	15.2–38.2	-	-	0.0001
Vegetables (g/d)	183	142–239	254	182–345	249	182–347	306	217–426	0.0001
Fruits (g/d)	181	99–311	206	114–312	213	128–320	275	151–422	0.0001
Beer (ml/d)	124	20–228	82	20–228	124	3–228	20	3–124	0.0001
Wine (ml/d)	17.5	8.8–53.8	17.5	8.8–98.8	17.5	8.8–53.8	8.8	1.3–53.8	0.0001
Fructose-rich drinks (ml/d)^5^	78.2	40.8–135.6	69.6	21.6–125.0	97.2	36.0–134.4	96.8	36.0–123.0	0.4
Coffee (ml/d)	477	95–502	272	84–665	477	152–625	84	4–477	0.0001
Tea (ml/d)	475	475–855	475	150–855	475	82–855	82	2–475	0.0001

	Mean	SD	Mean	SD	Mean	SD	Mean	SD	
Energy (kJ/d)	9248	2348	9042	2320	9096	2371	8001	2339	<0.0001
Protein (% of energy)	15.5	2.4	14.0	2.4	13.0	1.9	12.8	1.8	<0.0001
Dairy protein (% of energy)	3.6	1.5	3.6	1.8	3.8	1.7	-	-	0.5
Soy protein (% of energy)	0.1	0.5	0.7	1.1	0.8	0.9	2.6	1.6	<0.0001
Fat (% of energy)	31.8	5.6	31.3	6.2	30.9	6.0	29.4	7.4	<0.01
Carbohydrates (% of energy)	51.0	6.4	53.3	7.6	54.6	7.4	57.4	7.2	<0.0001
Total sugar (% of energy)	24.4	5.8	25.4	6.6	25.5	5.8	25.5	7.9	0.4
Alcohol (g/d)	14.7	16.5	15.4	18.4	15.2	17.3	10.6	16.2	0.0001
Englyst fibre (g/d)	18.8	7.1	22.6	7.6	23.0	7.5	27.3	8.6	<0.0001
Calcium (mg/d)	1043	310	1110	392	1126	385	569	209	<0.0001
Vitamin C (mg/d)	114	49	126	59	134	74	155	71	<0.0001

1 Differences in means, medians and proportions were assessed using ANOVA, Kruskal-Wallis tests and χ^2^ tests, respectively.

2 Data are missing for one or more participants: 30 for BMI and 4 for dairy milk.

3 The numbers of current smokers (current light+current heavy) are shown; the p-value corresponds to differences in proportions between all smoking categories (never, former, current light and current heavy) by diet group.

4 Total meat comprises red meat, processed meat, liver and poultry.

5 Fructose-rich drinks comprise fruit juice and sugar sweetened soft drinks.

**Table 2:** Characteristics and intake of certain foods and nutrients by diet group among women.

	Meat eaters (n = 256)		Fish eaters (n = 257)		Vegetarians (n = 255)		Vegans (n = 255)		
Characteristics	Mean	SD	Mean	SD	Mean	SD	Mean	SD	p^1^
Age (years)	40.4	11.1	40.7	10.7	40.4	10.8	40.2	11.4	>0.9
BMI (kg/m^2^)^2^	23.7	3.6	22.3	2.7	22.8	3.8	21.8	3.0	<0.0001
Current smoking (n)^3^	21		23		16		17		0.3

	Median	IQR	Median	IQR	Median	IQR	Median	IQR	
Total meat (g/d)^4^	61.8	35.4–96.5	-	-	-	-	-	-	-
Fish (g/d)	34.9	23.4–55.9	23.6	12.9–39.5	-	-	-	-	0.0001
Eggs (g/d)	7.0	3.5–21.5	7.0	3.5–21.5	7.0	3.5–21.5	-	-	0.6
Dairy milk (g/d)^2^	293	146–439	293	146–439	293	146–439	-	-	0.5
Dairy yoghurt (g/d)	22.7	10.1–63.0	35.3	10.1–71.8	35.3	10.1–101.0	-	-	0.2
Dairy cheese (g/d)	15.2	6.6–30.6	23.0	15.2–38.2	27.5	15.2–40.4	-	-	0.0001
Vegetables (g/d)	250	192–331	255	189–353	279	219–375	311	237–410	0.0001
Fruits (g/d)	230	158–366	276	173–421	248	168–411	267	155–452	0.2
Beer (ml/d)	2.9	2.9–40.3	20.2	2.9–40.3	2.9	2.9–40.3	2.9	2.9–40.3	0.02
Wine (ml/d)	17.5	8.8–53.8	53.8	8.8–98.8	17.5	1.3–53.8	8.8	1.3–53.8	0.0001
Fructose-rich drinks (ml/d)^5^	59.2	23.6–122.4	54.0	15.6–122.4	93.6	24.4–122.4	56.4	19.2–122.4	0.06
Coffee (ml/d)	475	29–512	192	15–488	203	15–502	84	4–477	0.0001
Tea (ml/d)	475	150–855	475	150–855	475	27–855	190	2–475	0.0001

	Mean	SD	Mean	SD	Mean	SD	Mean	SD	
Energy (kJ/d)	8139	2056	7603	2080	7940	2118	7076	2096	<0.0001
Protein (% of energy)	17.0	2.8	15.0	2.1	14.1	2.1	13.3	2.2	<0.0001
Dairy protein (% of energy)	3.7	1.7	4.2	2.0	4.3	2.1	-	-	<0.0001
Soy protein (% of energy)	0.3	0.6	0.9	1.0	1.0	1.0	3.0	1.9	0.0001
Fat (% of energy)	31.2	5.9	29.8	6.1	30.2	6.3	29.0	6.9	0.001
Carbohydrates (% of energy)	52.0	6.5	54.9	6.6	56.0	6.6	58.9	7.6	<0.0001
Total sugar (% of energy)	25.9	6.2	27.6	6.6	27.5	6.7	26.3	8.8	0.01
Alcohol (g/d)	7.9	9.9	9.4	12.6	8.0	10.4	5.6	9.2	0.0001
Englyst fibre (g/d)	19.9	6.7	21.9	8.3	23.0	7.7	25.7	8.8	<0.0001
Calcium (mg/d)	979	315	1034	349	1081	372	570	233	<0.0001
Vitamin C (mg/d)	142	70	149	76	154	76	161	85	0.03

1 Differences in means, medians and proportions were assessed using ANOVA, Kruskal-Wallis tests and χ^2^ tests, respectively.

2 Data are missing for one or more participants: 34 for BMI and 4 for dairy milk.

3 The numbers of current smokers (current light+current heavy) are shown; the p-value corresponds to differences in proportions between all smoking categories (never, former, current light and current heavy) by diet group.

4 Total meat comprises red meat, processed meat, liver and poultry.

5 Fructose-rich drinks comprise fruit juice and sugar sweetened soft drinks.

In men, there were significant differences between the diet groups in mean BMI; meat eaters had the highest BMI followed by vegetarians, fish eaters and vegans (lower by 2.5 kg/m^2^) (Table 1). In men, the diet groups also varied significantly in their intake of most food groups. Among the three diet groups consuming dairy products, meat eaters had a slightly higher intake of milk and the lowest intake of cheese. The vegans had the highest intake of vegetables and fruits, whereas the meat eaters had the lowest intake of these foods. Vegans had the lowest intake of beer, wine, coffee and tea. The four diet groups also differed significantly in their nutrient intakes. Meat eaters had the highest intake of energy and percent energy from protein but the lowest intake of dietary fibre. Vegans had the highest percent energy from soy protein and carbohydrates and the highest intake of dietary fibre and vitamin C, whereas they had the lowest alcohol intake, percent energy from fat and a markedly lower intake of calcium (approximately half that of the other diet groups).

In women, the pattern of differences between the diet groups was in general similar to that observed in men. In women however, the meat eaters had the highest intake of fish and the lowest percent energy from dairy protein and sugar; fish eaters had the highest intake of beer but the intakes of milk (among the three diet groups consuming dairy products) and fruit did not differ significantly by diet group (Table 2).

The correlations between dietary and non-dietary variables and uric acid concentrations are shown in Table 3 and were in general low (|r|<0.3). There was a significant positive correlation between uric acid concentrations and BMI (r = 0.28 and 0.26 in men and women, respectively) and a significant inverse correlation with percent energy from dairy protein (r = −0.10 and −0.11 in men and women, respectively) and calcium intake in both men and women (r = −0.17 and −0.16, respectively). After excluding the vegans from the correlation between uric acid concentrations and calcium intake, the correlation was attenuated and was no longer statistically significant in men (r = −0.03 and −0.11 in men and women, respectively). In men, there were significant positive correlations between uric acid concentrations and intakes of beer, alcohol and energy from soy protein. In women, uric acid concentrations and age were significantly positively correlated, whereas the uric acid concentrations were inversely correlated with intake of dairy milk. Smoking status was not associated with uric acid concentrations in either men or women (p = 0.7 and 0.2 in men and women, respectively).

**Table 3:** Partial correlation coefficients between serum uric acid concentrations and characteristics, food intake and nutrient intake adjusted for age and BMI.

	Men (n = 640)		Women (n = 989)	
Characteristics	Partial correlation^1^	p	Partial correlation^1^	p
Age (years)	−0.06	0.14	0.08	0.01
BMI (kg/m^2^)	0.28	<0.0001	0.26	<0.0001
Total meat (g/d)^2^	0.07	0.36	0.10	0.11
Fish (g/d)^3^	0.02	0.71	0.06	0.19
Eggs (g/d)^4^	−0.03	0.47	0.02	0.65
Dairy milk (g/d)^4^^,^ 5	−0.03	0.57	−0.08	0.04
Dairy yoghurt (g/d)^4^	−0.06	0.23	−0.06	0.11
Dairy cheese (g/d)^4^	−0.06	0.17	−0.01	0.81
Vegetables (g/d)	0.08	0.05	0.02	0.46
Fruits (g/d)	0.06	0.12	0.04	0.27
Beer (ml/d)^6^	0.15	0.0006	0.03	0.48
Wine (ml/d)^6^	0.02	0.69	0.05	0.13
Fructose-rich drinks (ml/d)^7^	0.02	0.68	0.05	0.15
Coffee (ml/d)	−0.04	0.30	−0.01	0.65
Tea (ml/d)	0.02	0.63	−0.05	0.12
Energy (kJ/d)	−0.05	0.25	−0.02	0.57
Protein (% of energy)	−0.06	0.14	−0.06	0.05
Dairy protein (% of energy)^4^	−0.10	0.04	−0.11	0.004
Soy protein (% of energy)	0.12	0.002	−0.01	0.75
Fat (% of energy)	−0.08	0.05	0.02	0.45
Carbohydrates (% of energy)	<0.01	0.92	−0.04	0.23
Total sugar (% of energy)	−0.02	0.70	−0.05	0.10
Alcohol (g/d)^6^	0.13	0.002	0.06	0.09
Englyst fibre (g/d)	0.06	0.10	0.03	0.36
Calcium (mg/d)	−0.17	<0.0001	−0.16	<0.0001
Calcium (mg/d) excluding vegans^4^	−0.03	0.47	−0.11	0.004
Vitamin C (mg/d)	0.07	0.07	0.04	0.23

1 Adjusted for age (20–29; 30–39; 40–49; 50–59; ≥60 years) and BMI (<20; 20–<22.5; 22.5–<25; 25–<27.5; ≥27.5 kg/m^2^).

2 In meat eaters only (163 men and 245 women). Total meat comprises red meat, processed meat, liver and poultry.

3 In meat and fish eaters only (322 men and 491 women).

4 In meat eaters, fish eaters and vegetarians only (478 men and 739 women).

5 Missing data for 4 men and 4 women.

6 In alcohol consumers only (drinking >0.4 g alcohol per day; 535 men and 797 women).

7 Fructose-rich drinks comprise fruit juice and sugar sweetened soft drinks.

**Figure 1:**
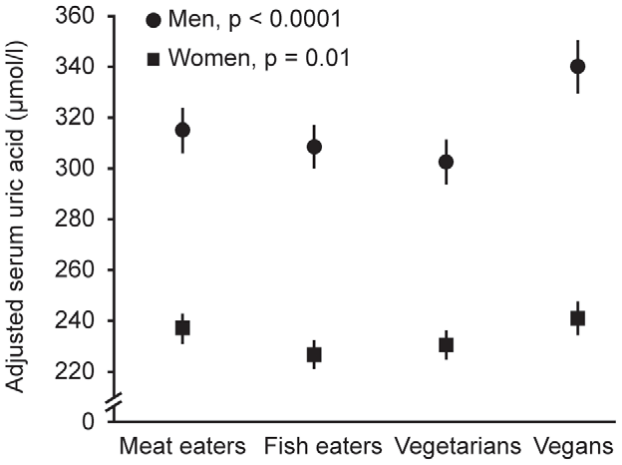
Adjusted serum uric acid concentrations by diet group and sex. The figure shows the adjusted means and 95% CIs of serum uric acid concentrations in meat eaters (163 men and 245 women), fish eaters (159 men and 246 women), vegetarians (156 men and 248 women) and vegans (162 men and 250 women). Serum uric acid concentrations were adjusted for age (20–29; 30–39; 40–49; 50–59; ≥60 years), alcohol intake (sex-specific fifths), BMI (<20; 20–<22.5; 22.5–<25; 25–<27.5; ≥27.5 kg/m^2^) and calcium intake (sex-specific fifths).

The results in Table 4 and Figure 1 show concentrations of serum uric acid by diet group for men and women separately. The age and alcohol adjusted concentration of uric acid was approximately 35% higher in men than in women but differences in uric acid concentrations between the diet groups followed a similar pattern in men and women. Vegans and meat eaters had the highest concentrations of uric acid, whereas fish eaters and vegetarians had the lowest concentrations of uric acid. After additional adjustment for BMI and calcium intake, the differences in concentration of uric acid became more pronounced in men; vegans had a significantly higher mean concentration than each of the other three diet groups. The largest difference in uric acid concentration for men was between vegans and vegetarians; the uric acid concentration in vegans being on average 37.6 µmol/l (95% CI 22.6–52.6) (12%) higher than in vegetarians. For women, the differences between the diet groups were slightly attenuated after the additional adjustment, and vegans and meat eaters had the highest concentrations of uric acid. The largest difference in mean uric acid concentration was 13.8 µmol/l (95% CI 4.2–23.4) (6%) between vegans and fish eaters.

**Table 4:** Mean (95% CI) concentrations of serum uric acid by diet group among men and women.

	Meat eaters	Fish eaters	Vegetarians	Vegans	p
**Men**					
n	163	159	156	162	
Adjusted serum uric acid (µmol/l)^1^	322.8 (314.1–331.5)	306.5 (297.7–315.3)	301.3 (292.4–310.2)	336.0 (327.1–344.8)	<0.0001
Adjusted serum uric acid (µmol/l)^2^	315.0 (306.1–324.0)	308.8 (299.9–317.6)	302.7 (293.8–311.5)	340.2 (329.4–351.1)	<0.0001
**Women**					
n	245	246	248	250	
Adjusted serum uric acid (µmol/l)^1^	238.8 (233.0–244.7)	224.2 (218.3–230.0)	228.2 (222.4–234.0)	242.7 (236.8–248.6)	<0.0001
Adjusted serum uric acid (µmol/l)^2^	236.6 (230.7–242.4)	226.8 (220.9–232.6)	229.9 (224.1–235.8)	240.6 (233.8–247.4)	0.01

1 Adjusted for age (20–29; 30–39; 40–49; 50–59; ≥60 years) and alcohol intake (sex-specific fifths).

2 Adjusted for variables mentioned in ^1^ plus BMI (<20; 20–<22.5; 22.5–<25; 25–<27.5; ≥27.5 kg/m^2^) and calcium intake (sex-specific fifths).

## Discussion

The results of this cross-sectional analysis showed that vegans had the highest concentrations of uric acid followed by meat eaters, and that fish eaters and vegetarians had the lowest uric acid concentrations. These differences by diet group were more pronounced in men than in women.

The higher uric acid concentrations among vegans might be due to their lack of consumption of dairy products, which are thought to lower uric acid concentrations [11]. Also, the low calcium content of the vegan diet might contribute to higher uric acid concentrations. While no previous studies have compared uric acid concentrations in vegans with other diet groups, previous cross-sectional studies have shown an inverse association between the intake of dairy products and circulating concentrations of uric acid [9], [30]. Moreover, results from small (n≤158) intervention studies have shown lower circulating concentrations of uric acid after the consumption of dairy products [31], [32]. This could be due to the low purine content and increased excretion of uric acid and its precursor xanthine in response to the protein in dairy products [11], [32]. Dairy products are the primary source of calcium and in the current study calcium was significantly inversely correlated with uric acid concentration, though this was partly due to the low intake of calcium among vegans. This result is in accordance with other cross-sectional analyses showing inverse correlations between calcium intake and circulating concentrations of uric acid [33], [34]. A randomised controlled trial did not, however, show an effect of calcium supplement (either 600 or 1200 mg/d) on uric acid concentrations after two years of treatment compared to placebo [34]. The mean intake of calcium at baseline in this intervention study (867 mg/d) [35] was higher than that of vegans in the present study (570 mg/d). Thus, it is possible that calcium supplement could reduce uric acid concentrations in individuals with low habitual calcium intake. Therefore, the lack of dairy products and the low calcium intake in the vegan diet might to some extent account for the observed differences in concentrations of uric acid between vegans and the other three diet groups.

Soy, other legumes and some vegetables are rich in purines [36], but it is possible that purines of vegetable origin have a different effect on uric acid concentrations than those of animal origin due to the different bioavailability and types of purines from these sources [1]. In the present study, vegans had the highest intake of soy protein and a significant positive correlation was observed between uric acid concentrations and soy protein in men. However, in other studies, purine-rich vegetables [33] or soy [37] were not associated with circulating concentrations of uric acid.

The high purine content of meat might explain the lower concentration of uric acid in vegetarians and fish eaters who both exclude meat from their diet. In accordance with our results, a small (n = 45) study did not find a difference in uric acid concentrations between vegetarians and fish eaters [38]. Other small (n≤114) studies have compared the uric acid concentration in vegetarians and meat eaters (also referred to as omnivores in some studies). Some [12]–[14] but not all [39] studies support our finding of lower uric acid concentrations in vegetarians than in meat eaters. In general, larger differences between diet groups were found in the prior studies compared with the current study. This may partly be explained by some of these studies [12], [13] not adjusting for BMI, which in the present study was the strongest confounding factor. The finding of similar uric acid concentrations in meat eaters and vegetarians in one of the prior studies [39] could be due to the fact that vegans (n = 6) were included in the vegetarian group (n = 31), which may have led to an overestimation of uric acid concentration among vegetarians.

The present study has some limitations. The finding of weak correlations between dietary variables and serum uric acid concentrations might be due to measurement error in the assessment of dietary intake [40] or because the FFQ measures usual diet over the past year, whereas uric acid concentration might only reflect a short period of time prior to blood collection [32]. We also performed a large number of statistical tests for correlations of serum uric acid with dietary and non-dietary variables, thus some of the significant correlations may be due to chance. Finally, some individuals with gout might have changed diet group, which could have led to reverse causation in this cross-sectional analysis. However, the prevalence of gout is low in individuals under the age of 50 (≤2.2% and ≤0.6% in men and women, respectively [41]), which was the age of most participants. Reverse causality is thus unlikely to account for the differences in uric acid concentration observed between diet groups. We were not able to exclude participants with gout because such information was not available.

As other studies have reported [14], [30], our results showed higher concentrations of uric acid in men than in women and we also observed more pronounced differences between diet groups in men than in women. Estrogens affect the renal excretion of uric acid positively; this might explain the sex difference in uric acid concentrations, but the mechanisms are not well understood [42]. Why the relationship between diet group and uric acid concentration is stronger in men than in women is not known. However, women might be less sensitive to the effect of diet on uric acid concentrations, possibly due to estrogens, but this needs investigation.

In conclusion, the results of the present study show that individuals consuming a vegan diet had the highest serum concentrations of uric acid compared to meat eaters, fish eaters and vegetarians, especially among men. Vegetarians and individuals who eat fish but no meat had the lowest serum uric acid concentrations. These findings highlight the importance of distinguishing between vegans and vegetarians in future studies of diet groups and uric acid concentrations. The potential effects on circulating uric acid concentrations of excluding dairy products and of low intake of calcium in the vegan diet deserves further investigation, as does the influence of different types and quantities of purines in the diet.
